# Autosomal Dominant Hypophosphatemic Rickets: A Case Report and Review of the Literature

**DOI:** 10.3390/ijerph18168771

**Published:** 2021-08-19

**Authors:** Chiara Mameli, Arianna Sangiorgio, Valeria Colombo, Mirko Gambino, Luigina Spaccini, Elisa Cattaneo, Gian Vincenzo Zuccotti

**Affiliations:** 1 Department of Pediatrics, V. Buzzi Children’s Hospital, Università di Milano, Via Lodovico Castelvetro, 32, 20154 Milan, Italy; arianna.sangiorgio@unimi.it (A.S.); valeria.colombo2@unimi.it (V.C.); mirko.gambino@unimi.it (M.G.); gianvincenzo.zuccotti@unimi.it (G.V.Z.); 2 Clinical Genetics Service, V. Buzzi Children’s Hospital, Università di Milano, Via Lodovico Castelvetro, 32, 20154 Milan, Italy; luigina.spaccini@asst-fbf-sacco.it (L.S.); elisa.cattaneo@asst-fbf-sacco.it (E.C.)

**Keywords:** rickets, hypophosphatemia, children, fibroblast growth factor 23 gene

## Abstract

Autosomal dominant hypophosphatemic rickets (ADHR) is an extremely rare form of genetic rickets caused by mutations in the fibroblast growth factor 23 gene. ADHR is characterized by hypophosphatemia secondary to isolated renal phosphate wasting. Only a few cases of ADHR have been reported in the literature to date. We describe the case of a 17-month-old girl who presented with severe failure to thrive (length: −4.08 standard deviation (SD), weight: −2.2 SD) and hypotonia. Hypophosphatemia, decreased tubular phosphate reabsorption (69%), and rachitic lesions were found. Genetic analysis showed the heterozygous variant c.536G>A (NM_020638.3:c.536G>A) in exon 3 of the FGF23 gene, leading to the diagnosis of ADHR. She was treated with phosphate salts and oral alfacalcidol. After 4 years of treatment, at 5 years of age, the patient’s ADHR resolved spontaneously. Considering the lack of knowledge regarding ADHR, we reviewed the literature to describe the features of this rare and poorly understood disease. Eleven ADHR pediatric cases have been described thus far, with cases tending to be more common in females than males. Similar to the general population, two groups of patients with ADHR can be described depending on the mutations present: patients with an R179 and R176 mutation have early-onset of disease and higher frequency of rickets, and a milder and late-onset of disease, respectively. Symptoms and disease severity may fluctuate. Spontaneous remission may occur during the pediatric age.

## 1. Introduction

Rickets is a systemic disease that occurs during developmental ages. It originates from an abnormal differentiation and maturation of chondrocytes, resulting in a lack of mineralization of the growth plate cartilage and bone deformity [1,2]. The most common form of rickets is secondary to vitamin D deficiency; apart from this, there are rare genetic conditions involving different bone homeostasis pathways, whose clinical presentations are very similar [1,2].

Among the forms of genetically determined rickets, hypophosphatemic rickets (HR) is the most commonly diagnosed [1,3]. HR includes hereditary hypophosphatemic rickets with hypercalciuria, X-linked hypophosphatemic rickets (XLHR, 1 in 20,000 births), autosomal dominant hypophosphatemic rickets (ADHR), and autosomal recessive hypophosphatemic rickets (ARHR).

HR can be caused both by loss- or gain-of-function mutations.

ARHR and XLHR are caused by loss-of-function mutations in the genes *DMP1/ENPP1* and *PHEX,* respectively, which encode for proteins involved in FGF23 production in osteocytes. Their loss-of-function increases FGF23 production, leading to hypophosphatemia. In Raine syndrome, phosphorylation of FGF23 is altered by a loss-of-function mutation in the extracellular protein kinase FAM20C, which causes reduced cleavage of FGF23, leading to hyperphosphaturia with a risk of rickets and/or osteomalacia [2].

ADHR (OMIM phenotype number #193100) is an extremely rare hereditary disorder caused by heterozygous point mutations in fibroblast growth factor 23 (FGF23) which is actively involved in phosphorous homeostasis [4]. FGF23 is a hormone produced by osteocytes that decreases phosphoremia, acting on both kidney and intestine phosphate absorption. In the kidney, it decreases the number of sodium-dependent phosphate transport proteins (NPT2A and NPT2C), decreasing phosphate renal tubular reabsorption. Likewise, FGF23 reduces intestinal phosphate absorption by enhancing *CYP24A1*, which encodes for the 24-hydroxylase that is responsible for 1.25 (OH)_2_D metabolic inactivation [2]. These mutations result in enhanced FGF23 bioactivity and lead to isolated renal phosphate wasting, hypophosphatemia, and impaired bone mineralization [3,5,6].

To the best of our knowledge, 49 cases of ADHR have been reported thus far [7,8,9,10,11,12,13,14,15,16].

The onset of ADHR is highly variable in terms of age presentation and clinical features. During childhood, the presentation is almost indistinguishable from the XLHR phenotype. The first manifestations occur around the age of walking, with progressive limb deformities, *coxa vara* or *genu valgu/varum*, skeletal pain, craniosynostosis, abnormal gait, and decreased growth velocity leading to disproportionate short stature [8,9,10,11,12,13]. The onset during adolescence or adulthood is characterized by bone pain, weakness, pseudofractures, but no deformity. Due to the rarity of ADHR, little is known about the prognosis. Symptoms and disease severity may fluctuate. Patients with the childhood-onset form may have postpubertal spontaneous remission with the resolution of hyperphosphaturia [8,9,10,11,12,13]. Moreover, the management of this form of rickets is challenging because of the lack of specific guidelines for ADHR.

This article aims to report a pediatric case of an early-onset ADHR with a severe phenotype and a prepubertal spontaneous resolution. We also review the pediatric cases of ADHR described in the literature so far to help clinicians in the management of this rare disease.

## 2. Case Report

A 17-month-old girl was referred to our endocrinology clinic with a history of failure to thrive since weaning at 6 months of age.

She was born at 38 weeks from an uncomplicated pregnancy, firstborn of healthy, non-consanguineous parents. Her birth weight was 2.8 kg (−0.46 standard deviations (SD) according to INeS charts) [17], her length was 48 cm (−0.21 SD—INeS charts), and her head circumference was 33 cm (−0.33 SD—INeS charts). The perinatal period was unremarkable. She had normal growth and motor development until 7-months-old, when she was unable to sit. Due to progressive delay in motor skills and the onset of trunk and limb hypotonia, she was hospitalized at the age of 15 months. The neurological evaluation confirmed the presence of hypotonia with hypotrophic muscles; cranial nerve function and tendon reflexes were normal. Complete blood count, thyroid function, lactate/pyruvate ratio, screening for celiac disease, electromyography, nerve conduction studies, and magnetic resonance imaging of the brain were normal. No causes of hypotonia were identified and she was discharged with a diagnosis of “hypotonic syndrome and motor delay”.

Our first examination was at 17 months of age. She weighed 7.8 kg (−2.2 SD—WHO standards) and was 68.5 cm long (−4.08 SD—WHO standards). She had a large anterior fontanelle, primary dentition erupted and appeared normal, but rachitic bracelet, rachitic rosary and *coxa vara* were noted.

**Figure 1:**
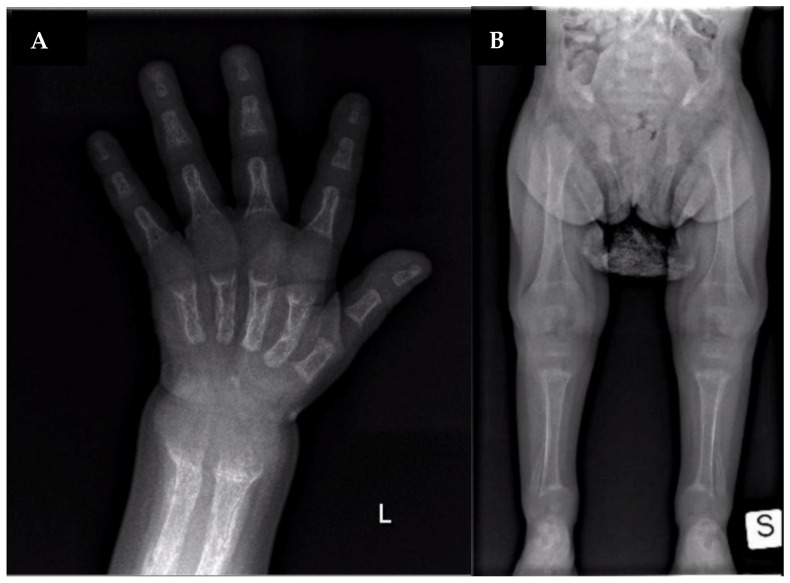
X-ray at diagnosis. (**A**): lateral widening of metaphyseal region; bone demineralization; fractures of metacarpus, radio, and ulna. (**B**): lateral widening of metaphyseal region; bowed fibula and tibia; bone demineralization; fractures of femur and fibula.

Laboratory analysis showed hypophosphatemia (1.3 mg/dL, normal values 4–7 mg/dL), increased alkaline phosphatase (ALP) activity (1173 U/L, normal values 140–400 U/L) and normocalcemia (9.2 mg/dL, normal values 8.8–10.8 mg/dL). 1,25-dihydroxy-vitamin D was normal (25 pg/mL, normal values 25–86.5 pg/mL), 25-hydroxy-vitamin D was 17 ng/mL (normal values >30 ng/mL) and parathormone (PTH) was slightly increased (96 pg/mL, normal values 15–65 pg/mL). Decreased tubular phosphate reabsorption (69%) was found, without hypercalciuria. The skeleton X-ray showed rib ends expansion (“rachitic rosary”), cupping and fraying of the metaphyseal regions, a diffusely reduced bone mineralization with periosteal reaction, and multiple fractures in various stages of healing (Figure 1). Body MRI excluded tumor-induced osteomalacia.

The next-generation sequencing (NGS) showed a de novo heterozygous variant c.536G>A (NM_020638.3:c.536G>A) in exon 3 of the FGF23 gene (the analysis of mutation segregation in the parents was negative), which lead to the diagnosis of ADHR.

This mutation located in site R179 is associated with an earlier onset of disease and a more severe phenotype compared to mutations located in position R176 [13].

At 23 months of age, the patient started oral alfacalcidol and oral phosphate. The dosage regimen was the same proposed for the treatment of X-linked HR [2,18,19], given the lack of treatment guidelines for this disease, as mentioned above. Cholecalciferol was also added to treat vitamin D insufficiency.

Moreover, considering low ferritin levels, oral iron supplementation was introduced based on the observation that iron supplementation may reduce phosphate loss in ADHR patients [7].

After 19 months of therapy, we reported a complete normalization of biochemical parameters and an improvement in muscle tone; at 3 years of age, she started walking. Growth velocity did not improve until the age of 4, when she had a catch-up growth. Leg X-ray showed an increase in bone mineralization and an improvement of the rachitic lesions, *genu valgum* deformity persisted.

**Figure 2:**
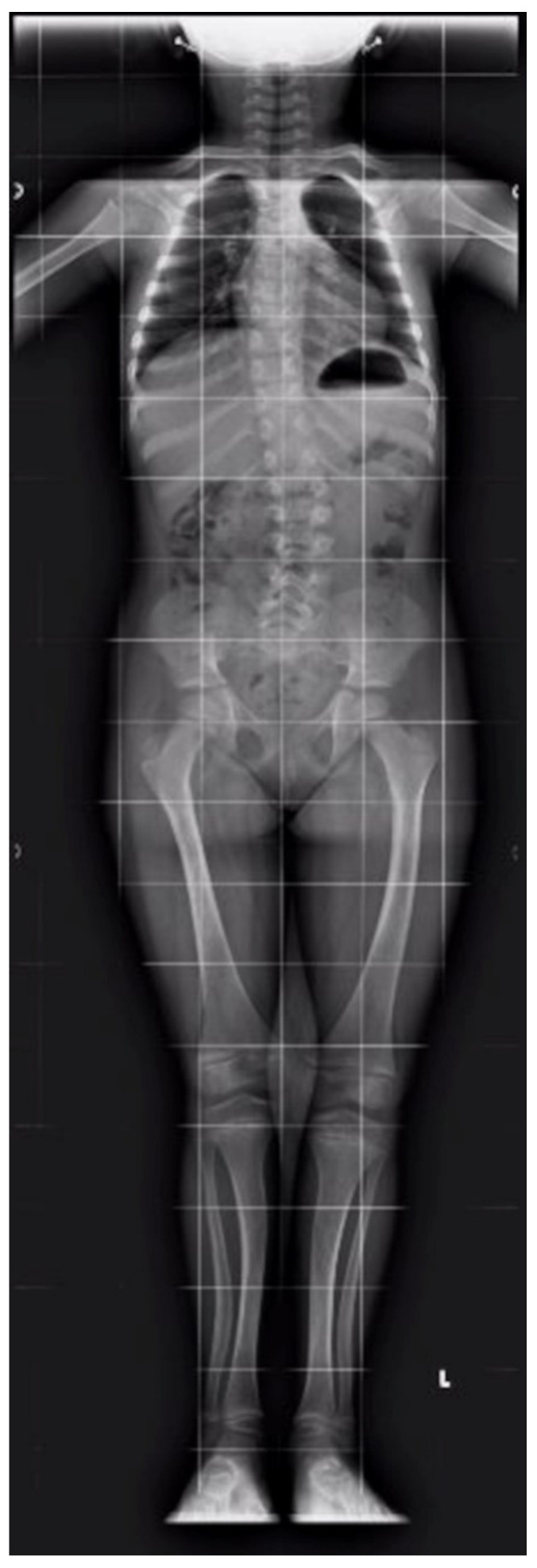
X-ray at the end of therapy.

At 5 and a half years old, we observed a spontaneous resolution of the disease. Therapy with alfacalcidol and phosphate was stopped. The biochemical examinations showed normal values of serum phosphate, calcium, creatinine, urinary calcium/urinary creatinine ratio, PTH, and 25OH-Vitamin D up to the age of 6 years and 6 months. Limbs X-ray showed an improvement of *genu valgum* and *cubitus varus*. No additional rachitic rosary nor remodeling rib lesions were present (Figure 2).

At the last follow-up evaluation (6 years and 6 months), growth parameters showed a constant improvement: the height was 107 cm (−2.28 SD), the weight 21.5 kg (−0.45 SD), with a growth velocity of 8.95 cm/y in 8 months (+3.35 SD).

## 3. Narrative Review of Literature and Discussion

ADHR is an extremely rare genetic disease. To the best of our knowledge, there are approximately 49 cases of patients with ADHR described in the literature, of which most are all diagnosed in adulthood [7,8,9,10,11,12,13,14,15,16]. The few pediatric cases reported in the literature are described in Table 1 [7,8,9,10,11,12,13].

Considering these few cases in the pediatric population, ADHR seems to be more common among females. Eight out of elevenchildren with ADHR are female (72%), including the index case.

As reported above, ADHR is caused by activating mutations in FGF23, which is actively involved in phosphorous homeostasis [2,18]. The FGF23 gene is composed of three exons on chromosome 12p13. Its protein, the fibroblast growth factor 23 (FGF23), plays a central role in the pathogenesis of hypophosphatemic rickets: high levels of active FGF23 cause hypophosphatemia, which leads to the onset of rachitic lesions. FGF23 protein exists in two forms: a full-length, mature form (FGF23^wt^, activated form), and a shorter form (FGF23^core^, inactive form). The shorter form is produced by proteolytic cleavage at the ^176^XXX^179^ site [20]. In fact, in the FGF23 protein, amino acids from 176 to 179 consist of a consensus sequence for proteolytic cleavage: Arg176-His177-Thr178-Arg179. Mutations located in one of these two arginines, such as R176Q (NP_065689.1:p.Arg176Gln), R179Q (NP_065689.1:p.Arg179Gln), R176W (NP 065689.1:p.Arg176Trp), and R179W (NP_065689.1:p.Arg179Trp), lead to a cleavage-resistant protein that remains in an active, intact form. This involves an exaggeration of the urinary excretion of phosphate [2,13].

Considering the 49 patients with ADHR (children and adults) described so far, 13 have the mutation NM_020638.3:c.526C>T, 19 the NM_020638.3:c.527G>A, six the NM_020638.3:c.535C>T, and 11 the NM_020638.3:c.536G>A [7,8,9,10,11,12,13,14,15,16]. The occurrence of mutations in the two sites R176Q/R176W and R179Q/R179W could represent mutational hotspots.

As shown in Table 1, all four genetic mutations associated with ADHR reported above are also represented in the pediatric population [13].

The clinical presentation of ADHR is heterogeneous. According to the age of presentation, two subgroups are described. One presents during childhood and is almost indistinguishable from X-linked phenotype. First manifestations occur around the age of walking, with rickets; progressive limb deformities, in particular *coxa vara* or *genu valgum/varum*; skeletal pain, craniosynostosis, abnormal gait, and decreased growth velocity leading to disproportionate short stature. Dental abnormality is common and may be the presenting complaint; tooth eruption is often delayed. The other subgroup presents during adolescence or adulthood with bone pain, weakness, and pseudofractures. Symptoms and disease severity may fluctuate and patients with childhood onset may have spontaneous remission with the resolution of hyperphosphaturia, especially after puberty [8,13].

Interestingly, in the index patient, the resolution was the most precocious reported so far and it occurred before puberty. Only patient VI-51, reported by Econs et al., did not present a resolution of the symptoms. Curiously, she is the oldest pediatric case reported until now [8]. Therefore, we could affirm that the natural history of ADHR is not predictable.

An interesting and recent paper by Liu et al. [13] shed new light on genotype-phenotype correlation between all ADHR patients, not only pediatric. The authors concluded that patients with the R179 mutations have an earlier onset of disease and are more likely to have rickets compared to patients with the R176 mutations. As shown in Table 1, the two mutations are almost equally represented in childhood-onset disease: 6/11 patients had an R176 mutation and 5/11 had an R179 mutation. Even though either mutation has an expression during childhood, according to Liu et al., pediatric patients with an R179 mutation have an earlier age of onset (17 months old vs. 60months old) and a more severe phenotype. All the children with an R179 mutation had rickets associated with important growth retardation. The only asymptomatic pediatric patient, whom the diagnosis was made due to the child’s symptomatic mother, had an R176 mutation.

The functional effects of the variants could be explained by different levels of resistance to the cleavage of FGF23 depending on the site of mutation involved [13]. Some in vitro studies have shown how FGF23 mutated in R179 is more resistant to cleavage compared to protein mutated in R176 [13].

The severity of growth impairment in ADHR is poorly defined. The anthropometric evaluations at diagnosis of ADHR were reported in only 6/11 described cases (Table 1). Based on these available data, the index case presented with the most severe growth impairment. Although the exact reason for this finding is unclear, we propose that it could partially be due to the delay in diagnosis. The patient presented to our endocrinology clinic with the typical features reported for childhood onset XLHR, but because hypotonia was a significant symptom, prior to our referral, neurological investigation was performed. Therefore, we recommend checking at least the serum phosphorus level in patients presenting with poor growth and progressive delay of development, even without signs of rickets.

As symptoms, biochemical and radiological findings of ADHR are similar to those of XLHR [2]. Low serum phosphorus, decreased tubular maximum reabsorption of phosphate (TMP/GFR), and inappropriately low-normal circulating 1,25(OH)2D levels are typical biochemical findings. Serum calcium levels are normal or slightly decreased, urinary calcium levels are low, PTH is normal or slightly elevated, serum alkaline phosphatase activity is elevated in children, but less than in vitamin-D-deficiency rickets (Table 1). When available, serum FGF23 testing shows an increase over the normal level. All these findings may fluctuate alongside the disease severity [2].

Radiological signs in ADHR are those of common rickets, but less severe than those observed in vitamin-D-deficiency rickets. The most typical alterations are seen in metaphyses of rapid-growing bones, such as the distal femur, tibia, and radio-ulnar joint. In contrast to nutritional rickets, the bone has a mesh-like appearance with gross bone trabeculations, and the cortex is thicker [2].

Management of these patients is based on XLHR therapeutic strategy, as said before, because of the absence of specific guidelines for ADHR due to the limited literature available around this disease. The milestone of HR therapy is calcitriol and phosphate supplementation. Oral phosphorous supplementation requires multiple daily intakes because of its 4 h half-life and the indication for dosage varies between 40 and 60 mg/kg/day in three to five doses [17,18,19]. The daily dosage is adjusted to clinical and biochemical efficacy in terms of bone pain control, ALP levels, growth velocity, and leg bowing. The most frequent complication of HR therapy is secondary hyperparathyroidism [2,18,19].

Active vitamin D analogue (calcitriol or alfacalcidol) has to be titrated on growth velocity expected according to age (20–30 mcg/kg/day, two to three times a day). Nephrocalcinosis is the most common complication and needs to be screened [2,18,19].

Orthopedic and surgical management are recommended when leg bowing persists despite optimal medical treatment, preferably at the end of childhood.

Recently, in 2018, a new therapeutic strategy for XLHR has been opened with the approval of Burosumab from the Food and Drug Administration and the European Medical Agency. Burosumab is a monoclonal antibody inhibiting FGF23, acting in reducing phosphaturia. Safety and efficacy are optimal with significant improvement in Rickets Severity Score. Until now, no studies are available about the application of Burosumab in ADHR patient.

## 4. Conclusions

The rarity of ADHR is still the central element of disease management. Few cases have been described so far, most of which are in adulthood. Lacking information about natural history, prognosis and therapeutic efficacy make the management of this disease a real dare. Aware of the precious role of case reports and previous experience in other centers, we have reported our experience on a case of childhood-onset ADHR. Similar to the other pediatric cases described above, the index patient presented early with poor growth. She showed extremely severe clinical features, in some way overlapping the typically reported presentation of childhood-onset XLHR. The follow-up that we can describe is limited in time. Our purpose is to follow the patient through childhood and adolescence and to observe the possible fluctuation of the natural history of the index’s disease. Of course, the available knowledge about ADHR in the literature is still limited, as widely exposed. More studies and reports are needed in the future to better understand and treat ADHR, especially in children.

## Figures and Tables

**Table 1:** Pediatric cases of ADHR.

Paper [Ref].	Econs 1997 [8].	Econs 1997 [8].	Econs 1997 [8].	Kruse 2001 [9].	Negri 2004 [10].	Gribaa 2010 [11].	Gribaa 2010 [11].	Kapelari 2015 [7].	Sandal 2020 [12].	Liu 2019 [13].	Index Case
**Case report**	Case VI-51	Case VI-26	Case VI-5	Case#1	Case#2	Case V3	Case V4	Case 1	Case 1	Case 3-III1	Case 1
**Sex**	F	M	M	F	F	F	M	F	F	F	F
**Genetic mutation**	R176Q NM_020638.3:c.527G>A rs104894347	R176Q NM_020638.3:c.527G>A; rs104894347,	R176Q NM_020638.3:c.527G>A rs104894347	R179Q NM_020638.3:c.536G>A; rs193922702	R179Q NM_020638.3:c.536G>A; rs193922702	R176W NM_020638.3:c.526C>T rs754201217	R176W NM_020638.3:c.526C>T rs754201217	R179Q NM_020638.3:c.536G>A; rs193922702	R179W NM_020638.3:c.535C>T; rs28937882	R176Q NM_020638.3:c.527G>A rs104894347	R179Q NM_020638.3:c.536G>A; rs193922702
**Age of onset**	14.5 y	14 m	19 m	11 m	8 m	3 y	5 y	26 m	2 y	NA	17 m
**Age of diagnosis**	14.5 y	14 m	19 m	11 m	8 m	3 y	5 y	26 m	13 y	2 y	19 m
**H at diagnosis**	N/A	50° to 5° ple	N/A	3° ple	3° ple	N/A	N/A	79 cm (−2.82 SDS)	142 cm (−2.68 Z-score)	NA	68.5 cm (−4.08 SD)
**W at diagnosis**	N/A	N/A	N/A	10° ple	N/A	N/A	N/A	9500 g (−2.12 SDS)	44.2 kg (−0.49 Z-score)	NA	7.8 kg (−2.2 SD)
**Ca at diagnosis**	8.9 mg/dL (nv 8.8–10.8)	9.2 mg/dL (nv 8.8–10.8)	9.5 mg/dL (nv 8.8–10.8)	2.53 mol/L (nv 2.44–2.7)	9.5 mg/dL (nv 8.8–10.8)	8.82 mg/dL (nv 8.8–10.8)	9.62 mg/dL (nv 8.8–10.8)	2.44 mmol/L (nv 2.3–2.7)	9.65 mg/dL (nv 8.8–10.8)	2.51 mmol/L (nv 2.3–2.7)	9.2 mg/dL (nv 8.8–10.8)
**P at diagnosis**	1.2 mg/dL (nv 4–7)	2.1–3.3 mg/dL (nv 4–7)	2.4 mg/dL (nv 4–7)	1.39 mmol/L (nv 1.64–2.58)	3 mg/dL (nv 4–7)	2.79 mg/dL (nv 4–7)	3.41 mg/dL (nv 4–7)	0.63 mmol/L (nv 1.1–1.95)	1.4 mg/dL (nv 4–7)	2.11 mmol/L	1.3 mg/dL (nv 4–7)
**ALP at diagnosis**	N/A	36.5 Bodansky U (nv < 15)	N/A	860 U/L (nv 200–600)	1755 U/L (nv < 640)	190 U/L	N/A	731 U/L (nv 200–600)	934 IU/L	205 U/L	1173 U/L (nv 140–400)
**PTH at diagnosis**	N/A	49 µL/Eq-mL (nv < 57)	N/A	3.8 pmol/L (nv 1.1–5.8)	45 pg/mL (nv 15–65)	N/A	N/A	37.3 pg/mL (nv 10–55)	185 pg/mL (nv 22–84)	NA	96 pg/mL (nv 15–65)
**TRP at diagnosis** **(nv 85–97%)**	85%	N/A	53%	47.5%	N/A	N/A	N/A	75.6%	NA	NA	69%
**25-OH D at diagnosis**	N/A	N/A	N/A	N/A	16 ng/mL (nv > 30)	N/A	N/A	139.2 nmol/L (nv > 50)	60 ng/mL (nv > 30)	NA	17 ng/mL (nv > 30)
**FGF23 at diagnosis**	N/A	N/A	N/A	N/A	N/A	N/A	N/A	N/A	NA	31.8 pg/mL	NA
**Ferritin at diagnosis**	N/A	N/A	N/A	N/A	N/A	N/A	N/A	5 μg/L (nv > 30)	NA	NA	NA
**Clinical features**	Bone pain, osteomalacia ankle soreness, low back pain, fatigue, pseudofractures	Rachitic lesions at the wrists and knees, growth retardation	Genu valgum, decreased energy, moderately severe rickets	Rachitic rosary, metaphyseal widening, fraying and cupping of the ulna and the distal femur	Genu varum, growth retardation	Bone deformities, dental hypoplasia, frontal bossing, short stature, pectus carinatum, anterior bowing of both legs, pelvis retroversion	Bone deformities, dental hypoplasia, frontal bossing, short stature, pectus carinatum, anterior bowing of both legs	Genu varu, severe rickets, waddling gait	Rickets, short stature, pain in spine/hips/legs and difficulty in walking, kyphoscoliosis, proximal muscle weakness in all four limbs, and genu varus, deformity of bilateral lower limbs	None	Hypotonia, Rickets, growth retardation
**Treatment**	Vitamin D 50.000–100.000 U/day	Vitamin D 25.000–50.000 U/day, then vitamin D 5–25.000 U/day (vitamin toxicity)	Vitamin D 30.000–50.000 U/day At 11 ys calcitriol 0.25–0.5 mcg twice daily	Calcitriol 0.125 mcg/day Oral phosphate 220–400 mg/day	Oral phosphate + Calcitriol (doses not available)	Oral phosphate (doses not available)	Oral phosphate (doses not available)	Oral phosphate 64 mg/kg/day Alfacalcidol 20 ng/kg/day At 8 ys iron sulfate solution	Vitamin D and calcium till 5 y of age. Since age of 13: Phosphate 60 mg/kg/d in four divided doses and calcitriol 60 ng/kg/d in three divided doses for 6 months	NA	Oral alfacalcidol (0.020 mcg/kg/day) and oral phosphate (30 mg/kg/day, divided in 3 doses)
**Symptoms Resolution**	No	Yes	Yes	Yes	Yes	Yes	Yes	Yes	Yes	NA	Yes
**Age at last follow-up visit** **(H/W if available)**	22 ys H: 163.1 cm W: 65.9 kg	20 ys	19.75 ys H 178.8 cm	8 ys	3 ys 6 mo	9 ys H: 138 cm	12 ys H: 150 cm	11.5 y H: 125.3 cm (−3.23 SDS) W: 26.2 kg (−2.2 SDS)	13 y	NA	On going

ALP: alkaline phosphatase; Ca: calcium; CaU/CrU: urine calcium to creatinine ratio; F: female; FGF23: fibroblast growth factor 23; H: height; m: months; M: male, N/A: not available; nv: normal values; ple: percentile; P: phosphorus; PTH: parathyroid hormone; SDS: standard deviation score; T: time (months from diagnosis); TRP: tubular phosphorus reabsorption; U: unit; W: weight; y: years; 25-OH vit D: 25-hydroxyvitamin D.

## Data Availability

Individual participant data that underlie the results reported in this article, after de-identification will be available upon request.
